# Disseminated cryptococcosis in a geriatric man following high‐dose systemic steroid therapy for severe COVID‐19 pneumonia

**DOI:** 10.1002/jgf2.652

**Published:** 2023-09-21

**Authors:** Hinako Nawa, Dai Akine, Tomohiro Tamura, Koyumi Saito, Takayuki Kaburagi, Teppei Sasahara

**Affiliations:** ^1^ Division of Respiratory Medicine, Department of Medicine, Ibaraki Prefectural Central Hospital Ibaraki Cancer Center Kasama Japan; ^2^ Division of Infectious Diseases, Department of Medicine, Ibaraki Prefectural Central Hospital Ibaraki Cancer Center Kasama Japan; ^3^ Department of Infection and Immunity, School of Medicine Jichi Medical University Shimotsuke Japan; ^4^ Department of Dermatology, Ibaraki Prefectural Central Hospital Ibaraki Cancer Center Kasama Japan

**Keywords:** COVID‐19, disseminated cryptococcosis, high‐dose systemic steroid therapy

## Abstract

An 88‐year‐old man was treated with high‐dose systemic steroid therapy for COVID‐19 and idiopathic interstitial pneumonia months before admission to the hospital because of swelling and redness in his left arm. *Cryptococcus neoformans* was detected in his blood sample on day eight of admission, and despite antifungal therapy, he died on day 43. Clinicians should be vigilant about the risk of prolonged immunosuppression as a side effect of high‐dose systemic steroid usage for COVID‐19.

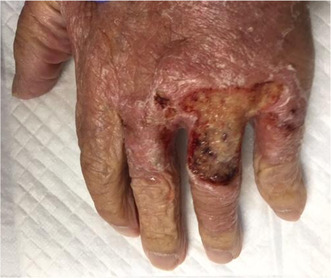

An 88‐year‐old man was hospitalized because of worsening of redness and swelling in his left arm. Eight months before admission, he was diagnosed with coronavirus disease 2019 (COVID‐19), and treated with methylprednisolone 500 mg for 6 days. Three months before admission, he was hospitalized for idiopathic interstitial pneumonia and was treated with 500 mg methylprednisolone for 3 days followed by oral prednisolone. One month before admission, he developed redness and swelling in his left arm (Figure 1). Despite antibacterial treatment, his condition did not improve. *Cryptococcus neoformans* was detected in his blood sample on day eight of admission. While his skin had no appropriate lesions to perform a skin biopsy, we considered the skin as the portal of entry for cryptococcus. Although we could not evaluate his spinal fluid, we decided to administer an antifungal regimen assuming that the patient had meningitis as a complication. Therefore, liposomal amphotericin B and flucytosine were initiated. On day 23, his skin lesions deteriorated, with ulcers on the dorsum of the right hand (Figure 2) and the appearance of internal hematocysts on the extensor aspect of the left forearm. The hematocyst aspirate was positive for cryptococcal antigens and showed yeast‐like fungi that stained with Grocott's stain. His general condition worsened, and he passed away on day 43 of hospitalization.

**FIGURE 1:**
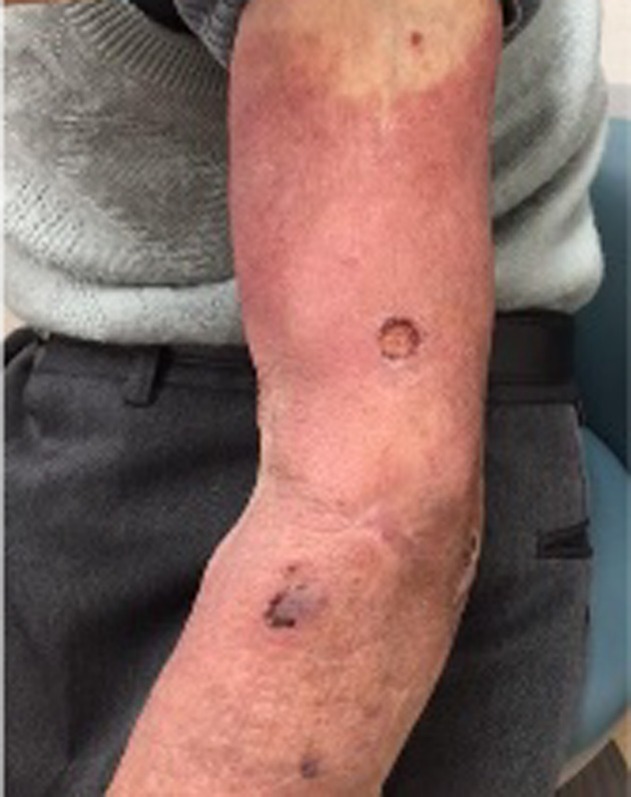
Redness and swelling were observed on the patient's left arm at the time of the initial examination.

**FIGURE 2:**
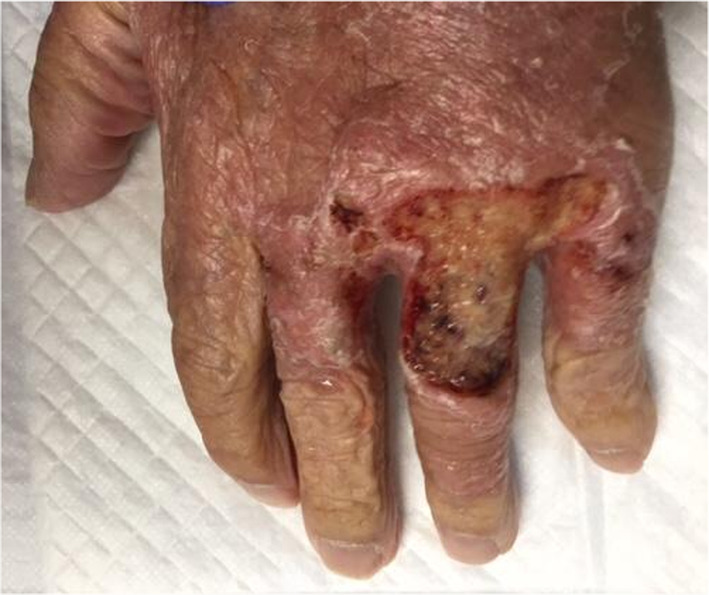
Ulcer formation was observed on the dorsum of the patient's right hand, as the skin lesions worsened despite appropriate antifungal treatment.

Corticosteroid use can cause cellular immunodeficiency and risk for invasive aspergillosis. Beardsley et al.^1^ reviewed the literature on cases of cryptococcal meningitis in non‐HIV patients and found that 23.2% of 164 patients were on corticosteroids during disease onset. Chen et al.^2^ reported a case–control study of patients with lupus nephritis who developed cryptococcal meningitis. They found that patients who developed the disease received an average of 27.5 ± 10.2 mg of steroids, whereas those who did not received an average of 15.5 ± 7.8 mg. Furthermore, 25% of patients who developed the disease received steroid pulse therapy within 1 year, while only 6.25% of those who did not develop the disease received steroid pulse therapy. Cumulative steroid use and a recent history of steroid pulse therapy may be associated with the development of cryptococcal infection.

In Japan, high‐dose systemic steroid therapy (HDST) is commonly used for severe lung diseases. Even COVID‐19 pneumonia was treated with HDST in the early phase of the pandemic.^3^ However, its effectiveness and safety have not been established. Two randomized controlled trials (RCTs) have reported on the efficacy of HDST for COVID‐19, but neither found any significance in adding HDST to standard therapy.^4^, ^5^

This case highlights the prolonged immunosuppressive condition caused by steroid use for the treatment of COVID‐19 and the importance of closely monitoring patients treated with steroids. The contribution of high‐dose systemic steroids and subsequent oral steroid administration to the development of disseminated cryptococcosis is still unclear in our patient; however, clinicians should remain vigilant of these risks in patients treated with steroids for COVID‐19.

## AUTHOR CONTRIBUTIONS

H.N. and D.A. contributed to the acquisition, analysis and interpretation of clinical data of the case. T.T. and K.S. contributed to the analysis and interpretation of clinical data. H.N., D.A. and T.S. wrote the manuscript. T.T., K.S. and T.K. reviewed and edited the manuscript. All authors revised and approved of the final version of the work.

## CONFLICT OF INTEREST STATEMENT

The authors have stated explicitly that there are no conflicts of interest in connection with this article.

## PATIENT CONSENT STATEMENT

We obtained informed consent from the patient's family for this case report.

## ETHICS STATEMENT

The study was approved by the Ethics Committee of Ibaraki Prefectural Central Hospital (approval number: 1203).
